# Angiotensin II Promotes White Adipose Tissue Browning and Lipolysis in Mice

**DOI:** 10.1155/2022/6022601

**Published:** 2022-06-27

**Authors:** Zhaohua Cai, Liang Fang, Yangjing Jiang, Min Liang, Jian Wang, Yejiao Shen, Zi Wang, Feng Liang, Huanhuan Huo, Changqing Pan, Linghong Shen, Ben He

**Affiliations:** ^1^Heart Center, Shanghai Chest Hospital, Shanghai Jiaotong University, Shanghai 200030, China; ^2^General Surgery Department, Shanghai Chest Hospital, Shanghai Jiaotong University, Shanghai 200030, China

## Abstract

Emerging evidence has revealed that all components of the renin-angiotensin system (RAS) are present in adipose tissue. Angiotensin II (Ang II), the major bioactive component of the RAS, has been recognized as an adipokine involved in regulating energy homeostasis. However, the precise role of Ang II in white adipose tissue (WAT) remodeling remains to be elucidated. In this present study, C57BL/C male mice were continuously infused with different doses of Ang II (1.44 mg/kg/d or 2.5 mg/kg/d) or saline for 2 weeks and treated with or without the Ang II type 1 receptor blocker valsartan. H&E staining and immunohistochemistry were conducted to investigate the white-to-brown fat conversion. The level of serum total cholesterol (TC), triglyceride (TG), low-density lipoprotein cholesterol (LDL-C), and high-density lipoprotein cholesterol (HDL-C) was measured. RNA sequencing was employed to explore the differentially expressed genes and their enriched pathways between control and Ang II groups. Our results showed that Ang II substantially resulted in loss of body weight and fat mass. Most importantly, Ang II treatment induced WAT browning in mice, which was partially attenuated by valsartan treatment. Furthermore, Ang II perturbed the serum lipid profiles. Ang II treatment elevated serum levels of TC, TG, LDL-C, and HDL-C in mice. Mechanistically, thermogenesis, cell respiration, and lipid metabolism-associated mRNAs showed significantly increased expression profiling in Ang II-treated WATs compared with control WATs. Moreover, we found that Ang II treatment enhanced AMPK phosphorylation in adipocytes. Therefore, Ang II promotes WAT browning and lipolysis via activating the AMPK signaling pathway.

## 1. Introduction

Adipose tissue can be mainly categorized into two phenotypically distinct types: white and brown adipose tissue (WAT and BAT, respectively). In contract to the notorious WAT that stores excess energy as triglycerides, BAT dissipates chemical energy in the form of heat by uncoupling mitochondrial respiratory chain and ATP synthesis [1]. Apart from the classical interscapular BAT, brown-like (also called beige or brite) adipocytes are also present in certain WAT depots. The development of beige adipocytes in WAT (so called “browning”) can be induced by chronic cold exposure [2–4], repeated injection of *β*3-adrenergic agonists [2], and some hormones (such as thyroid hormone T3 and fibroblast growth factor 21 (FGF21)) [5–7]. Emerging evidence suggests that beige adipocytes in WAT promote whole-body energy expenditure and confer beneficial effects on obesity and insulin resistance [8, 9].

WAT is not just a passive depot for lipid storage but also the largest endocrine organ secreting various adipokines, which play important roles in regulating energy metabolism in adipose tissue and whole body. Accumulating evidence demonstrates that adipokines also modulate WAT browning. For example, chronic cold exposure induces adiponectin accumulation in subcutaneous white adipose tissue (SWAT), which in turn enhances SWAT browning [10]. In addition, apelin which is an adipocyte-derived hormone enhances browning of white adipocytes [11, 12]. Angiotensin II (Ang II), the major bioactive component of the renin-angiotensin system (RAS), has been recognized as an adipokine which plays important roles in regulating energy homeostasis. Emerging evidence has revealed that all components of the RAS are present in adipose tissue, implying the involvement of local RAS in regulating adipose function. However, in vitro and in vivo studies examining the role of RAS in WAT function and remodeling have yielded conflicting results [13–16]. Tsukuda et al. found that Ang II type 1 receptor (AT1R) blockade enhanced WAT browning in mice [13]. Kim et al. demonstrated that AT1R antagonism by losartan stimulated WAT browning [14]. In addition, Than et al. revealed that Ang II type 2 receptor (AT2R) activation promotes WAT browning and brown adipogenesis [15]. This discrepancy strongly suggests the need for additional studies to address the role of Ang II in adipose tissue remodeling.

To explore the effect of Ang II on WAT function and remodeling, histopathological and transcriptomic approaches were employed in this study. Our findings demonstrate that Ang II promotes white adipose tissue browning and lipolysis via activating the AMPK signaling pathway.

## 2. Materials and Methods

### 2.1. Animal Experiments

All animal experiments were performed in accordance with the NIH guidelines for the Care and Use of Laboratory Animals. The study protocol was approved by the Committee on the Ethics of Animal Experiments of the Shanghai Jiaotong University School of Medicine. C57BL/6 male mice (8-12 weeks) were purchased from Shanghai Model Organisms Center, Inc. (Shanghai, China). C57BL/6 male mice were randomly assigned into two groups (vehicle group with saline and 2.5 mg/kg/d Ang II group) or four groups (vehicle group, 1.44 mg/kg/d Ang II group, 2.5 mg/kg/d Ang II group, and 2.5 mg/kg/d Ang II with valsartan group). The mice were infused with 1.44 mg/kg/d or 2.5 mg/kg/d Ang II (Sigma-Aldrich, Cat# A9525) for two weeks through subcutaneous osmotic minipumps (Model 2002, ALZA Scientific Products, Mountain View, CA, USA). These two doses of Ang II were chosen based on their well-known effects to induce vascular remodeling or abdominal aortic aneurysm and cardiac hypertrophy [17–19]. Saline-infused mice served as the controls. Valsartan (40 mg/kg/d) was administered daily via oral gavage for two weeks. The body weight and blood pressure of each mouse were measured weekly.

### 2.2. Blood Pressure Measurement

Blood pressure was measured by the tail-cuff method using a sphygmomanometer (BP-2010A, Softron, Tokyo, Japan) according to the manufacturer's protocol while the mice were conscious.

### 2.3. Tissue Collection and Processing

Mice were euthanized by inhalation of 5% isoflurane and cervical dislocation where appropriate. Tissue sections were stored at -80°C for RNA extraction or western blotting. Tissue sections for pathological diagnosis or immunohistochemistry were fixed in 10% neutral buffered formalin. Paraffin-embedded adipose tissue sections were cut at 8 *μ*m thickness.

### 2.4. Histology and Immunohistochemistry

Paraffin-embedded tissue sections were stained with hematoxylin and eosin (H&E) according to standard protocol [20]. All images were recorded using an Olympus digital camera (Tokyo, Japan). For immunohistochemical staining, paraffin-embedded tissue sections were deparaffinized, rehydrated, and subjected to antigen retrieval. Endogenous peroxidase activity was blocked using 0.3% H_2_O_2_ for 20 min. Tissue sections were blocked with normal goat serum (Biogenex, Cat# HK112-9K) and incubated with primary antibodies against uncoupling protein 1 (Ucp1) (Abcam, Cat# ab10983, 1 : 500). Goat anti-rabbit/mouse IgG (DAKO, Cat# K4061, ready-to-use) was used as secondary antibodies. The reaction was visualized using DAB (DAKO, Cat# K3468), and sections were counterstained with hematoxylin.

### 2.5. Serum Analysis

Total cholesterol (TC), triglyceride (TG), low-density lipoprotein cholesterol (LDL-C), and high-density lipoprotein cholesterol (HDL-C) in mouse serum were determined by an automatic biochemical analyzer following the manufacturer's instruction.

### 2.6. Preadipocyte Culture

Mouse 3T3-L1 preadipocytes were purchased from American Type Culture Collection (ATCC, Manassas, USA) and cultured according to the manufacturer's instruction. Primary white preadipocytes, isolated from mouse WAT, were also used. After digestion with 0.2% collagenase and centrifugation (1500 rpm, 5 min), the stromal vascular fraction/preadipose cells from WAT were isolated as described before [11, 21]. Preadipose cells were seeded in 6-well plates and cultured with the basal growth medium (DMEM supplemented with 10% 10% fetal bovine serum (FBS), 100 U/mL penicillin, and 100 mg/mL streptomycin). For adipocyte differentiation, fully confluent cells (defined as day 0) were treated with induction medium (basal growth medium supplemented with 1 *μ*M insulin, 1 *μ*M dexamethasone, and 50 *μ*M isobutylmethylxanthine) for 3 days. At day 3, the cells were incubated with differentiation medium (basal growth medium supplemented with 1 *μ*M insulin and 1 nM 3,5,3′-triiodothyronine). The medium was replenished every other day for the next 5 days. During the adipocyte differentiation, cells were treated with or without Ang II (0.5 *μ*M, 1 *μ*M, and 2 *μ*M).

### 2.7. RNA Sequencing

Total RNA extraction and RNA-seq analysis were performed by Shanghai Majorbio Bio-pharm Technology Co., Ltd. (Shanghai, China). Briefly, total RNA was extracted from the SWAT of the mice treated with saline or 2.5 mg/kg/d Ang II using Trizol reagent (Invitrogen, Carlsbad, CA, USA). Purification of mRNA was achieved by Oligo (dT)-attached magnetic beads. RNA-seq libraries were sequenced in a single lane using an Illumina NovaSeq 6000 sequencer (Illumina, San Diego, CA). The heat map was drawn using the pheatmap (v1.08) R package to exhibit fold changes of the gene expression levels in different samples. Gene Ontology (GO) and Kyoto Encyclopedia of Genes and Genomes (KEGG) analyses were conducted to evaluate the pathway enrichment.

### 2.8. Western Blotting

Total protein was prepared from cultured cells or mouse adipose tissues, and western blotting was performed as briefly described below. Proteins were quantified with a Pierce BCA Protein Assay Kit, separated by SDS-PAGE, and transferred to nitrocellulose membranes. Membranes were blocked with 5% nonfat milk dissolved in Tris-buffered saline with Tween 20 (TBST) at 37°C for 1 h and incubated with primary antibodies against AMP-activated protein kinase alpha (AMPK*α*) (Cell Signaling Technology, Cat# 2532, 1 : 1000), phospho-AMPK*α* (Thr172) (Cell Signaling Technology, Cat# 2531, 1 : 1000), *β*-actin (Santa Cruz, Cat# sc-47778, 1 : 1000), or GAPDH (Santa Cruz, Cat# sc-32233, 1 : 1000) at 4°C overnight. After incubation with horseradish peroxidase-conjugated secondary antibodies at 37°C for 1 h, proteins were detected using Pierce ECL Western Blotting Substrate and quantified using Quantity One 4.4.0 software (Bio-Rad, Hercules, CA, USA).

### 2.9. Statistical Analysis

Unpaired two-tailed Student's *t*-tests were used to calculate significant differences between two groups. Multiple comparison correction analysis was performed using one-way ANOVA with Tukey's post hoc HSD test. *p* < 0.05 was considered statistically significant. All values are expressed as mean ± SD.

## 3. Results

### 3.1. Ang II Reduces Body Weight and Fat Mass in Mice

C57BL/6 male mice were infused with saline or Ang II at a dose of 2.5 mg/kg/d for 2 weeks. Ang II infusion increased systolic and diastolic blood pressure (Supplementary Figure S1) and caused cardiac hypertrophy, as evidenced by significant increases in absolute and relative heart weights after Ang II infusion (Supplementary Figures S2(b) and S2(e)). Although the body weights from two groups were comparable prior to Ang II infusion, we found that Ang II treatment significantly reduced body weight (Supplementary Figure S2(a)). Moreover, the absolute and relative weights of subcutaneous white adipose tissue (SWAT) and epididymal white adipose tissue (EWAT) were significantly reduced in Ang II-infused mice (Supplementary Figures S2(c), S2(d), S2(f), and S2(g)).

**Figure 1:**
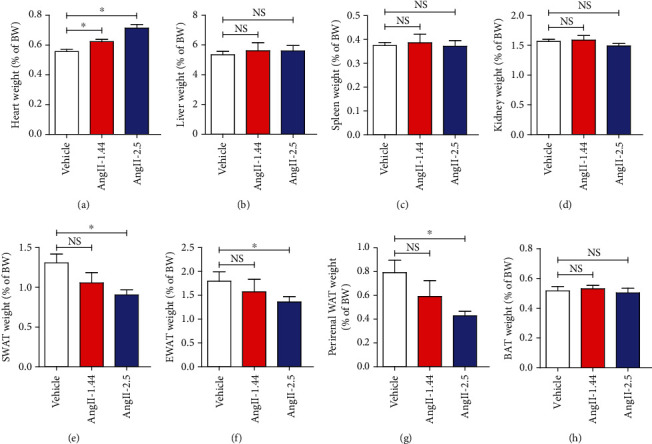
Ang II dose-dependently reduces fat mass in mice. Relative weights of (a) heart, (b) liver, (c) spleen, (d) kidney, (e) subcutaneous white adipose tissue (SWAT), (f) epididymal white adipose tissue (EWAT), (g) perirenal white adipose tissue (perirenal WAT), and (h) brown adipose tissue (BAT) in C57BL/6 mice infused with different doses of Ang II (1.44 mg/kg/d and 2.5 mg/kg/d) or saline for 2 weeks.

To confirm these results, C57BL/6 mice were further treated with different doses of Ang II (1.44 mg/kg/d and 2.5 mg/kg/d for 2 weeks). Treatment of mice with Ang II dose dependently increased absolute and relative heart weights (Supplementary Figure S3(a) and Figure 1(a)), whereas there were no observable changes in liver, spleen, and kidney weights among saline and different doses of Ang II groups (Supplementary Figures S3(b)–S3(d) and Figures 1(b)–1(d)). Although 1.44 mg/kg/d Ang II seems to reduce the weights of SWAT, EWAT, and perirenal WAT, there was no significant difference between vehicle and 1.44 mg/kg/d Ang II groups (Supplementary Figures S3(e)–S3(g) and Figures 1(e)–1(g)). However, 2.5 mg/kg/d Ang II dramatically reduces the weights for SWAT, EWAT, and perirenal WAT (Supplementary Figures S3(e)–S3(g) and Figures 1(e)–1(g)), suggesting the effect of Ang II on WAT is dose-dependent. Of note, we found that Ang II did not affect BAT weights (Supplementary Figure S3(h) and Figure 1(h)). Collectively, these findings suggest that Ang II substantially results in loss of body weight and WAT mass.

### 3.2. Ang II Induces White Adipose Tissue (WAT) Browning

**Figure 2:**
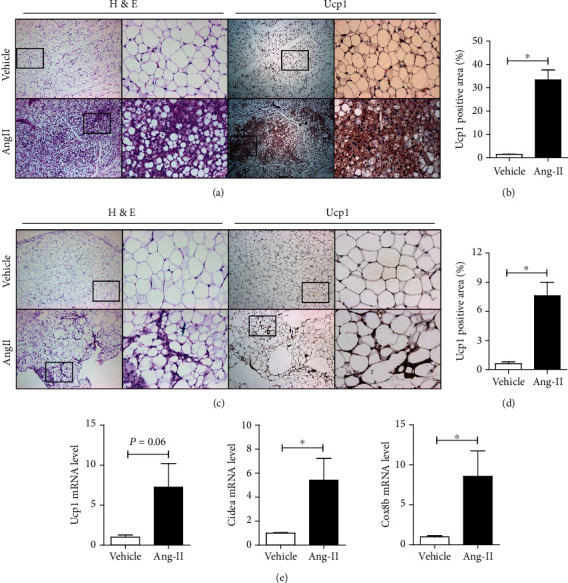
Ang II induces white adipose tissue (WAT) browning. (a) H&E and Ucp1 immunohistochemistry (IHC) staining in SWATs in mice infused with saline or Ang II (2.5 mg/kg/d) for 2 weeks. (b) Quantification of Ucp1-positive area in SWATs of mice from (a). (c) H&E and Ucp1 IHC staining in EWATs. (d) Quantification of Ucp1-positive area in EWATs. (e) Gene expression analysis of browning marker in EWATs.

To figure out the mechanisms underlying the adiposity-reducing effect of Ang II, we made a histological analysis of SWAT and EWAT from mice infused with Ang II or saline. H&E staining showed that the adipocyte sizes were smaller in Ang II-exposed SWAT compared with controls (Figure 2(a)). Most importantly, the SWAT of Ang II-treated mice contained widespread smaller and multilocular lipid droplets, indicative of brown-like/beige adipocytes arise after Ang II treatment (Figure 2(a), left panel). Immunohistochemical analysis showed that Ang II treatment markedly increased the expression of Ucp1, a specific marker for BAT, in SWAT (Figure 2(a) (right panel) and Figure 2(b)), further confirming the SWAT browning induced by Ang II.

Previous studies have shown that the appearance of brown-like/beige adipocytes is observed mainly in SWAT. Very few of these beige adipocytes can be detected in the visceral fat [2, 22]. Notably, we found that epididymal adipose tissue showed numerous new cells after Ang II treatment (Figure 2(c), left panel). Unlike beige adipocytes in SWAT, these new cells had fewer lipid droplets (Figure 2(c), left panel). Immunohistochemical analysis also showed increased Ucp1 expression in EWAT after Ang II treatment (Figure 2(c) (right panel) and Figure 2(d)). Moreover, quantitative real-time PCR analysis showed a consistent increase of browning markers, including Ucp1, Cidea, and Cox8b, in EWAT of Ang II-treated mice (Figure 2(e)). These results indicated that Ang II treatment promotes browning of SWAT and EWAT.

### 3.3. Activation of Lipolysis by Ang II Treatment

**Figure 3:**
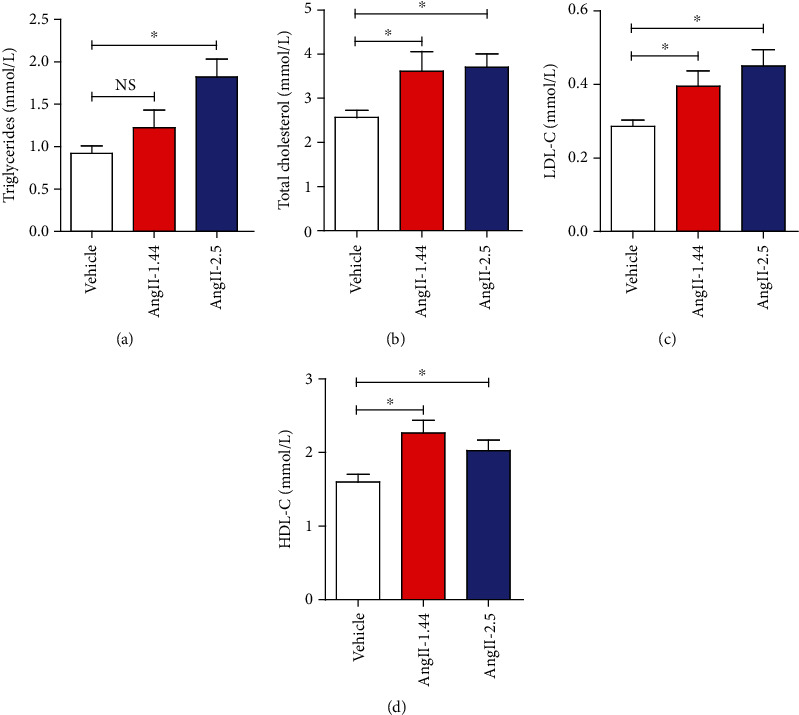
Activation of lipolysis by Ang II treatment. (a) Triglyceride, (b) total cholesterol, (c) low-density lipoprotein cholesterol (LDL-C), and (d) high-density lipoprotein cholesterol (HDL-C) were measured in C57BL/6 mice infused with different doses of Ang II (1.44 mg/kg/d and 2.5 mg/kg/d) or saline for 2 weeks.

To figure out whether the effect of Ang II on WAT will change the lipid metabolism in mice, we investigate serum lipid profiling of mice treated with Ang II or saline. We found that Ang II-infused mice exhibited perturbed serum lipid profiles (Figure 3). In detail, Ang II treatment significantly elevated serum levels of TG, TC, LDL-C, and HDL-C (Figures 3(a)–3(d)), contributing to hyperlipidemia in C57BL/6 mice. This result suggests that Ang II can lead to increased adipose tissue lipolysis.

### 3.4. The Effect of Ang II on WAT Browning Is Partially Mediated by Ang II Type 1 Receptor (AT1R)

**Figure 4:**
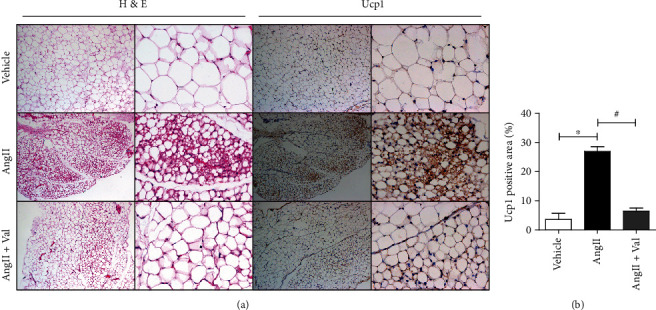
The effect of Ang II on WAT browning is partially mediated by Ang II type 1 receptor (AT1R). (a) H&E and Ucp1 immunohistochemistry (IHC) staining in SWATs of mice infused with saline or Ang II (2.5 mg/kg/d) for 2 weeks and treated with or without the Ang II type 1 receptor blocker valsartan. (b) Quantification of Ucp1-positive area in SWATs of mice from (a).

Ang II type 1 and type 2 receptors (AT1R and AT2R, respectively) are well known to be involved in the complex cardiovascular actions of Ang II [23]. To investigate whether the effects of Ang II on adipose tissues are mediated by its receptor, Ang II-infused mice were treated with the AT1R blocker valsartan. We found that valsartan treatment partially attenuated the WAT browning induced by Ang II (Figure 4), indicating that the effect of Ang II on WAT browning is mediated in part by AT1R.

### 3.5. Transcriptomics-Based Screening of Differential Gene Expression Profile

**Figure 5:**
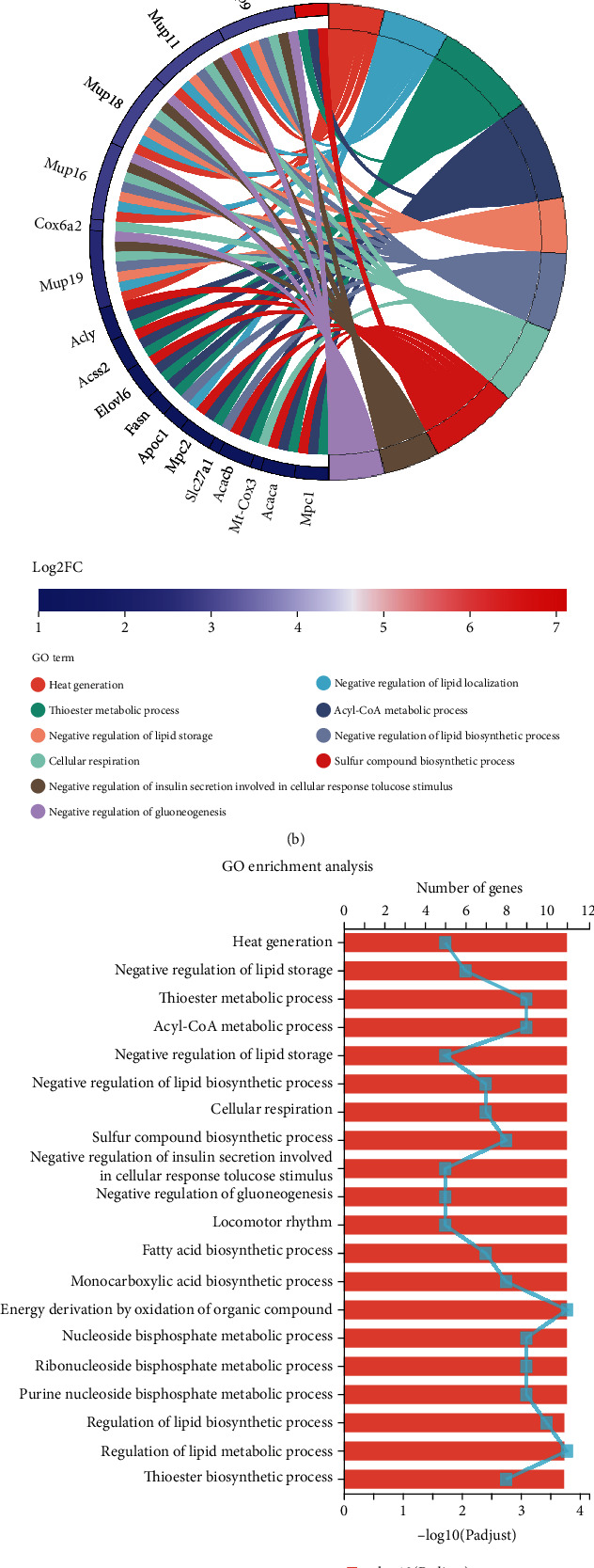
Transcriptomics-based screening of differential gene expression profile. (a) The heat map shows the upregulated and downregulated genes in SWATs from C57BL/6 mice infused with saline or Ang II (2.5 mg/kg/d) for 2 weeks. Each column represents an individual replicate, and *n* = 3 replicates for each group. Each row represents an individual gene. The color bar represents the *z*-score-transformed relative expression of normalized counts of genes that are upregulated and downregulated. (b) String diagram of differentially expressed genes (DEGs) based on GO functional enrichment analysis. (c) GO pathway analysis of DEGs between control and Ang II groups. (d) KEGG pathway analysis of DEGs between control and Ang II groups.

To explore the molecular mechanisms, we analyzed whole mRNA expression profiling in SWAT from mice infused with Ang II or saline. Ang II treatment resulted in noticeable alterations of gene expression patterns (Figure 5(a)). As expected, we found that Ucp1 is highly enriched in Ang II-treated SWAT (Figure 5(a)). Of note, multiple genes associated with thermogenesis (Cox8b, Adcy10, Klb, Cox6a2, Ucp1, mt-Co3, mt-Nd3, Cox7a1, Cpt1b, and Dio2), cell respiration (Cox6a2, mt-Co3, Mup11, Mup19, Mup18, Mup16, and Mup9), acyl-CoA metabolic process (Acaca, Acly, Mpc1, Fasn, Mpc2, Acss2, and Acacb), and lipid metabolism (Acaa1b, Echdc1, Acaca, Acly, Fasn, Agpat2, Fabp5, Acss2, Slc27a1, Thrsp, Aspg, Elov3, Apoc1, Elov6, Acacb, Adtrp, Cpt1b, and Acad11) were significantly increased in Ang II-treated SWATs compared with control SWATs, indicative of WAT browning and lipolysis after Ang II treatment (Figures 5(a) and 5(b)).

Moreover, the top 20 of pathway enrichment was classified based on GO and KEGG analyses. The GO analysis further confirmed the functional changes after Ang II treatment (Figure 5(c)). Furthermore, the KEGG analysis revealed that Ang II may regulate several pathways, including fatty acid metabolism, metabolic pathways, PPAR signaling pathways, AMPK signaling pathways, and thermogenesis (Figure 5(d)). These data provide a comprehensive insight into the transcriptomic modifications induced by Ang II and demonstrate that the AMPK signaling pathway might be a critical mediator in the biological process of Ang II-induced WAT thermogenesis.

### 3.6. Ang II Treatment Enhances AMPK Phosphorylation in Adipocytes

**Figure 6:**
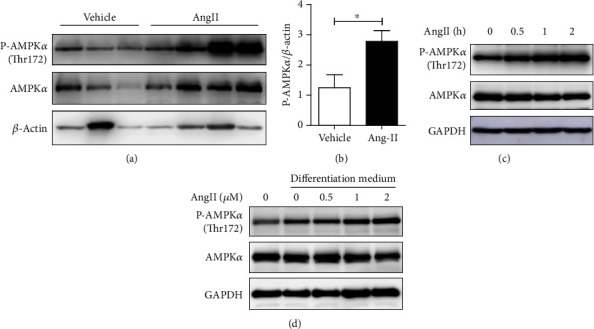
Ang II treatment enhanced AMPK*α* phosphorylation in adipocytes. (a, b) Western blot analyses assessing the protein expression levels of phospho-AMPK*α* (P-AMPK*α*) and AMPK*α* in WATs from C57BL/6 mice infused with saline or Ang II (2.5 mg/kg/d) for 2 weeks. (c) Western blot analyses assessing the protein expression levels of P-AMPK*α* and AMPK*α* in 3T3-L1 cells treated with 1 *μ*M Ang II for the indicated time. (d) Stromal vascular fraction (SVF)/preadipocytes were isolated from SWAT of C57BL/6 mice and cultured in differentiation medium with or without Ang II treatment. Western blot analyses assessing the protein expression levels of P-AMPK*α* and AMPK*α* in these cells.

As a well-known cellular sensor of energy homeostasis, AMPK has been strongly implicated in BAT function and lipid metabolism [24–26]. To further explore the molecular mechanisms underlying the effect of Ang II on WAT browning, we focused on the AMPK signaling pathway. We found that Ang II treatment enhanced AMPK*α* phosphorylation in vivo in mouse WAT (Figures 6(a) and 6(b)). In vitro experiment further indicated that AMPK*α* phosphorylation was increased after 3T3-L1 preadipocytes were treated with Ang II (Figure 6(c)). Moreover, we isolated the stromal vascular fraction (SVF) from mouse WAT and found increased activation of AMPK*α* after Ang II treatment during the differentiation process (Figure 6(d)). These results suggested that the AMPK signaling pathway was activated after Ang II treatment in adipocytes.

## 4. Discussion

Emerging evidence suggests adipokines secreted by adipose tissue act as the autocrine and paracrine signals to regulate its own browning [10–12]. Ang II has been recognized as an adipokine for its important roles in regulating energy homeostasis [27]. It has recently been shown that mouse and human preadipocytes can be differentiated into beige adipocytes after Ang II treatment in vitro [15]. However, the *in vivo* role of Ang II in WAT remodeling and lipid metabolism has not been well investigated. In this present study, we demonstrate that Ang II substantially reduces body weight and WAT mass, while increasing absolute and relative heart weights which indicates cardiac hypertrophy. Histopathological and transcriptomic approaches further indicate that Ang II, as an endocrine hormone supplying to adipose tissue via circulation, promotes adipose tissue browning and thermogenic gene expression in SWAT. This strongly suggests multiple organs or tissues of actions of Ang II throughout the body, including not only the heart but also the adipose tissue. It has been demonstrated that adipocytes express both Ang II receptors (AT1R and AT2R) [28]. In this study, we demonstrated that the effect of Ang II on WAT browning is mediated in part by AT1R.

Previous studies have indicated that WAT browning is associated with increased WAT lipolysis. For example, in adipose tissue, agonists of the *β*3-adrenergic receptor regulate WAT browning, lipolysis, and lipid oxidation [29]. Extensive burn injuries cause WAT browning and increase the lipolysis of WAT in clinical patients and mouse models [30–32]. In this present study, we found that Ang II treatment elevated serum levels of TC, TG, LDL-C, and HDL-C, contributing to hyperlipidemia in mice. Therefore, our findings support that Ang II can lead to augmented adipose tissue lipolysis.

In this study, in order to investigate the underlying molecular mechanism by which Ang II induces WAT browning, we analyzed differentially expressed genes (DEGs) between the control and Ang II-treated SWATs and determined the significant functions and pathways by KEGG. Fatty acid metabolism, metabolic pathways, PPAR signaling pathways, thermogenesis, insulin resistance, and AMPK signaling pathways include the most gene number among the significantly enriched pathways. It has been well described that AMPK functions as a crucial metabolic sensor and is strongly implicated in regulating lipid synthesis, lipolysis, and brown and beige adipose tissue function [24–26]. Our in vivo and in vitro results demonstrate that the AMPK signaling pathway is activated after Ang II treatment in adipocytes, suggesting that the AMPK signaling pathway might be a critical mediator in the biological process of Ang II-induced WAT thermogenesis. This is opposite to the observations in a previous study which demonstrated that increased systemic Ang II leads to body weight loss in mice and Ang II reduces AMPK phosphorylation and signaling leading to skeletal muscle wasting [33, 34]. Ang II might exert functions in various tissues through its different effects on AMPK signaling. Nevertheless, it is imperative to further investigate the precise role of AMPK signaling in Ang II-induced WAT browning and lipolysis in the future.

In conclusion, the present study demonstrates that besides its endocrine actions in the blood vessel, heart, and kidney, adipose tissue is also another target of Ang II, where it promotes WAT browning and lipolysis via activating the AMPK signaling pathway. The important effect of Ang II on adipose tissue provides insights into how Ang II can be an important mediator for the interplay between adipose tissue and vasculature.

## Data Availability

All data is available in the main text or the supplementary files.

## Supplementary Materials

Supplementary 1 Figure S1: Ang II infusion increased systolic and diastolic blood pressure in mice. (a, b) Blood pressure was measured using a noninvasive tail-cuff sphygmomanometer before and 1 week and 2 weeks after Ang II infusion. Quantification of systolic blood pressure (a) and diastolic blood pressure (b).

Supplementary 2 Figure S2: Ang II reduces body weight and fat mass in mice. (a) Quantification of body weight in C57BL/6 mice infused with saline or Ang II (2.5 mg/kg/d) for 2 weeks. (b–g) Absolute and relative weights of heart (b, e, respectively), subcutaneous white adipose tissue (SWAT) (c, f, respectively), and epididymal white adipose tissue (EWAT) (d, g, respectively) in C57BL/6 mice infused with saline or Ang II (2.5 mg/kg/d) for 2 weeks.

Supplementary 3 Figure S3: Ang II dose-dependently reduces fat mass in mice. (a–h) Absolute weights of heart (a), liver (b), spleen (c), kidney (d), subcutaneous white adipose tissue (SWAT) (e), epididymal white adipose tissue (EWAT) (f), perirenal white adipose tissue (perirenal WAT) (g), and brown adipose tissue (BAT) (h) in C57BL/6 mice infused with different doses of Ang II (1.44 mg/kg/d and 2.5 mg/kg/d) or saline for 2 weeks.

## References

[1] Nedergaard J., Golozoubova V., Matthias A., Asadi A., Jacobsson A., Cannon B. (2001). UCP1: the only protein able to mediate adaptive non-shivering thermogenesis and metabolic inefficiency. *Biochimica et Biophysica Acta*.

[2] Wang Q. A., Tao C., Gupta R. K., Scherer P. E. (2013). Tracking adipogenesis during white adipose tissue development, expansion and regeneration. *Nature Medicine*.

[3] Berry D. C., Jiang Y., Graff J. M. (2016). Mouse strains to study cold-inducible beige progenitors and beige adipocyte formation and function. *Nature Communications*.

[4] Oguri Y., Shinoda K., Kim H. (2020). CD81 controls beige fat progenitor cell growth and energy balance via FAK signaling. *Cell*.

[5] Fisher F. M., Kleiner S., Douris N. (2012). FGF21 regulates PGC-1*α* and browning of white adipose tissues in adaptive thermogenesis. *Genes & Development*.

[6] Abu-Odeh M., Zhang Y., Reilly S. M. (2021). FGF21 promotes thermogenic gene expression as an autocrine factor in adipocytes. *Cell Reports*.

[7] Johann K., Cremer A. L., Fischer A. W. (2019). Thyroid-hormone-induced browning of white adipose tissue does not contribute to thermogenesis and glucose consumption. *Cell Reports*.

[8] Harms M., Seale P. (2013). Brown and beige fat: development, function and therapeutic potential. *Nature Medicine*.

[9] Bartelt A., Heeren J. (2014). Adipose tissue browning and metabolic health. *Endocrinology*.

[10] Hui X., Gu P., Zhang J. (2015). Adiponectin enhances cold-induced browning of subcutaneous adipose tissue via promoting M2 macrophage proliferation. *Cell Metabolism*.

[11] Than A., He H. L., Chua S. H. (2015). Apelin enhances brown adipogenesis and browning of white adipocytes. *The Journal of Biological Chemistry*.

[12] Dodd G. T., Decherf S., Loh K. (2015). Leptin and insulin act on POMC neurons to promote the browning of white fat. *Cell*.

[13] Tsukuda K., Mogi M., Iwanami J. (2016). Enhancement of adipocyte browning by angiotensin II type 1 receptor blockade. *PLoS One*.

[14] Kim D. Y., Choi M. J., Ko T. K., Lee N. H., Kim O. H., Cheon H. G. (2020). Angiotensin AT_1_ receptor antagonism by losartan stimulates adipocyte browning via induction of apelin. *The Journal of Biological Chemistry*.

[15] Than A., Xu S., Li R., Leow M. K., Sun L., Chen P. (2017). Angiotensin type 2 receptor activation promotes browning of white adipose tissue and brown adipogenesis. *Signal Transduction and Targeted Therapy*.

[16] Cao X., Shi T. T., Zhang C. H. (2022). ACE2 pathway regulates thermogenesis and energy metabolism. *eLife*.

[17] Chen H. Z., Wang F., Gao P. (2016). Age-associated sirtuin 1 reduction in vascular smooth muscle links vascular senescence and inflammation to abdominal aortic aneurysm. *Circulation Research*.

[18] Wang Q., Ding Y., Song P. (2017). Tryptophan-derived 3-hydroxyanthranilic acid contributes to angiotensin II-induced abdominal aortic aneurysm formation in mice in vivo. *Circulation*.

[19] Shen Y., Wang X., Yuan R. (2021). Prostaglandin E1 attenuates AngII-induced cardiac hypertrophy via EP3 receptor activation and netrin-1upregulation. *Journal of Molecular and Cellular Cardiology*.

[20] Cui M., Cai Z., Chu S. (2016). Orphan nuclear receptor Nur77 inhibits angiotensin II-induced vascular remodeling via downregulation of *β*-catenin. *Hypertension*.

[21] Kim H. J., Cho H., Alexander R. (2014). MicroRNAs are required for the feature maintenance and differentiation of brown adipocytes. *Diabetes*.

[22] Seale P., Bjork B., Yang W. (2008). PRDM16 controls a brown fat/skeletal muscle switch. *Nature*.

[23] Mogi M., Iwai M., Horiuchi M. (2007). Emerging concepts of regulation of angiotensin II receptors: new players and targets for traditional receptors. *Arteriosclerosis, Thrombosis, and Vascular Biology*.

[24] Mottillo E. P., Desjardins E. M., Crane J. D. (2016). Lack of adipocyte AMPK exacerbates insulin resistance and hepatic steatosis through brown and beige adipose tissue function. *Cell Metabolism*.

[25] O'Neill H. M., Holloway G. P., Steinberg G. R. (2013). AMPK regulation of fatty acid metabolism and mitochondrial biogenesis: implications for obesity. *Molecular and Cellular Endocrinology*.

[26] Watt M. J., Steinberg G. R. (2008). Regulation and function of triacylglycerol lipases in cellular metabolism. *The Biochemical Journal*.

[27] Pahlavani M., Kalupahana N. S., Ramalingam L., Moustaid-Moussa N. (2017). Regulation and functions of the renin-angiotensin system in white and brown adipose tissue. *Physiology*.

[28] Cassis L. A., Police S. B., Yiannikouris F., Thatcher S. E. (2008). Local adipose tissue renin-angiotensin system. *Current Hypertension Reports*.

[29] Bachman E. S., Dhillon H., Zhang C. Y. (2002). betaAR signaling required for diet-induced thermogenesis and obesity resistance. *Science*.

[30] Sidossis L. S., Porter C., Saraf M. K. (2015). Browning of subcutaneous white adipose tissue in humans after severe adrenergic stress. *Cell Metabolism*.

[31] Patsouris D., Qi P., Abdullahi A. (2015). Burn induces browning of the subcutaneous white adipose tissue in mice and humans. *Cell Reports*.

[32] Barayan D., Vinaik R., Auger C., Knuth C. M., Abdullahi A., Jeschke M. G. (2020). Inhibition of lipolysis with acipimox attenuates postburn white adipose tissue browning and hepatic fat infiltration. *Shock*.

[33] Yoshida T., Tabony A. M., Galvez S. (2013). Molecular mechanisms and signaling pathways of angiotensin II-induced muscle wasting: potential therapeutic targets for cardiac cachexia. *The International Journal of Biochemistry & Cell Biology*.

[34] Tabony A. M., Yoshida T., Galvez S. (2011). Angiotensin II upregulates protein phosphatase 2C*α* and inhibits AMP-activated protein kinase signaling and energy balance leading to skeletal muscle wasting. *Hypertension*.

