# Supplementary figures and images for: Angiotensin II Promotes White Adipose Tissue Browning and Lipolysis in Mice
Source: Oxid Med Cell Longev. 2022 Jun 27;2022:6022601. doi: 10.1155/2022/6022601 (PMC9253869; doi:10.1155/2022/6022601)

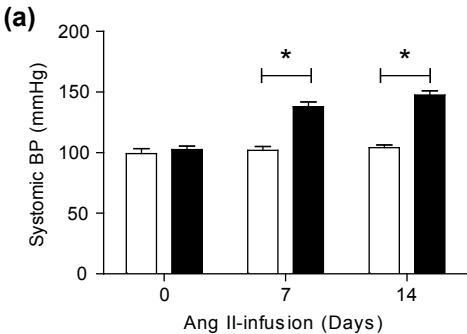

Vehicle

Ang II

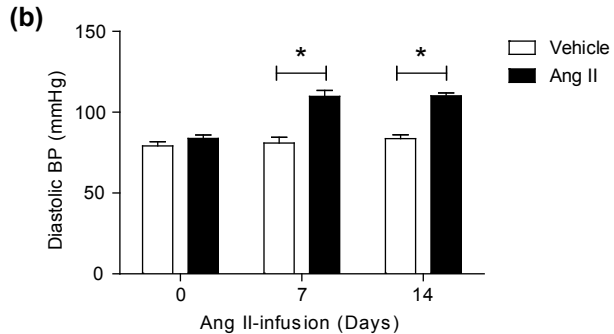

Vehicle

Ang II

Supplement: Supplementary 1 — Figure S1: Ang II infusion increased systolic and diastolic blood pressure in mice. (a, b) Blood pressure was measured using a noninvasive tail-cuff sphygmomanometer before and 1 week and 2 weeks after Ang II infusion. Quantification of systolic blood pressure (a) and diastolic blood pressure (b). [file 6022601.f1.pdf]

**(a)**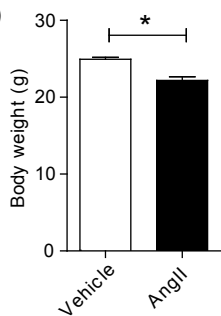**(b)**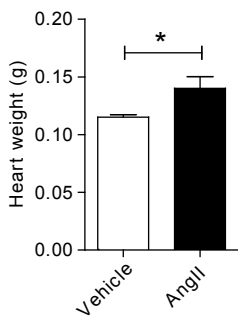**(c)**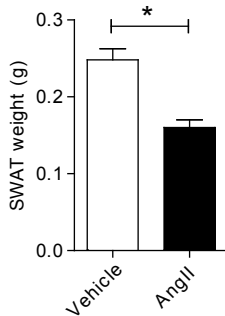**(d)**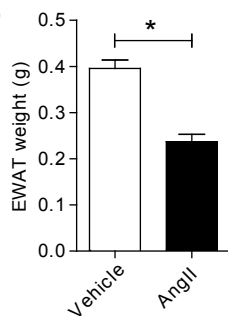**(e)**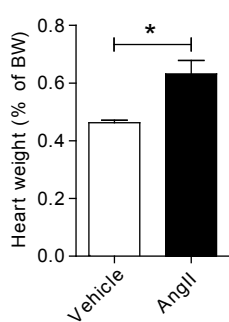**(f)**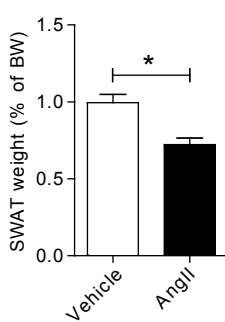**(g)**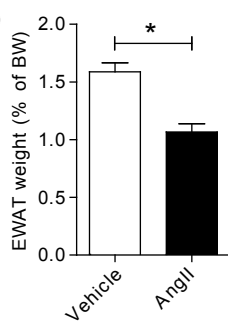

Supplement: Supplementary 2 — Figure S2: Ang II reduces body weight and fat mass in mice. (a) Quantification of body weight in C57BL/6 mice infused with saline or Ang II (2.5 mg/kg/d) for 2 weeks. (b–g) Absolute and relative weights of heart (b, e, respectively), subcutaneous white adipose tissue (SWAT) (c, f, respectively), and epididymal white adipose tissue (EWAT) (d, g, respectively) in C57BL/6 mice infused with saline or Ang II (2.5 mg/kg/d) for 2 weeks. [file 6022601.f2.pdf]

**(a)**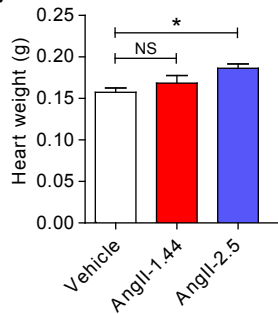**(b)**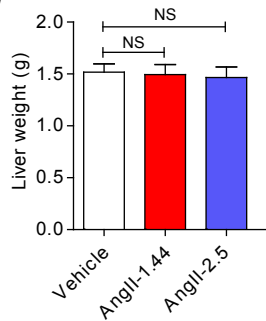**(c)**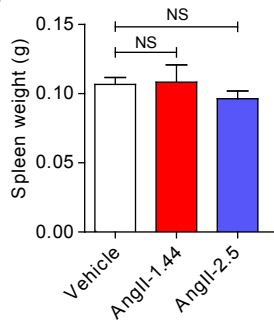**(d)**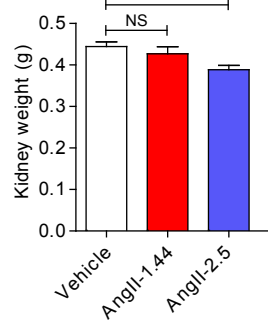**(e)**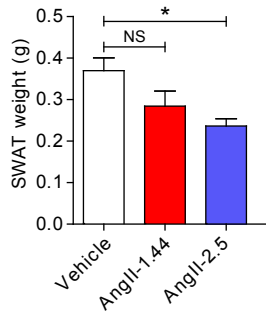**(f)**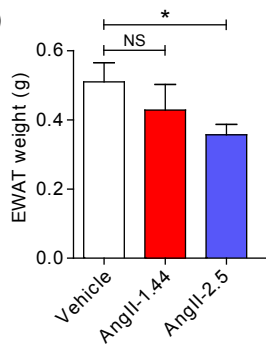**(g)**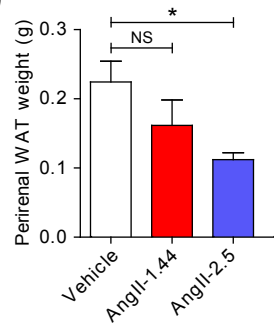**(h)**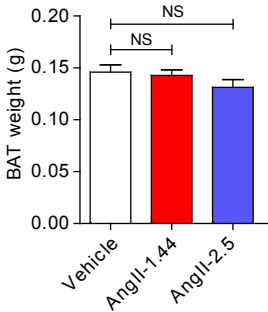

Supplement: Supplementary 3 — Figure S3: Ang II dose-dependently reduces fat mass in mice. (a–h) Absolute weights of heart (a), liver (b), spleen (c), kidney (d), subcutaneous white adipose tissue (SWAT) (e), epididymal white adipose tissue (EWAT) (f), perirenal white adipose tissue (perirenal WAT) (g), and brown adipose tissue (BAT) (h) in C57BL/6 mice infused with different doses of Ang II (1.44 mg/kg/d and 2.5 mg/kg/d) or saline for 2 weeks. [file 6022601.f3.pdf]
